# Phylogenomic Analysis Substantiates the *gyrB* Gene as a Powerful Molecular Marker to Efficiently Differentiate the Most Closely Related Genera *Myxococcus*, *Corallococcus*, and *Pyxidicoccus*

**DOI:** 10.3389/fmicb.2021.763359

**Published:** 2021-10-11

**Authors:** Yang Liu, Tao Pei, Shuoxing Yi, Juan Du, Xianjiao Zhang, Xiaoqin Deng, Qing Yao, Ming-Rong Deng, Honghui Zhu

**Affiliations:** ^1^Guangdong Provincial Key Laboratory of Microbial Culture Collection and Application, Key Laboratory of Agricultural Microbiomics and Precision Application, Ministry of Agriculture and Rural Affairs, State Key Laboratory of Applied Microbiology Southern China, Guangdong Open Laboratory of Applied Microbiology, Guangdong Microbial Culture Collection Center (GDMCC), Institute of Microbiology, Guangdong Academy of Sciences, Guangzhou, China; ^2^Guangdong Province Key Laboratory of Microbial Signals and Disease Control, College of Horticulture, South China Agricultural University, Guangzhou, China

**Keywords:** phylogenomic analysis, novel marker *gyrB* gene, species identification, differentiate, the most closely related genera

## Abstract

Rapid and accurate strain identification of the most closely related genera *Myxococcus*, *Corallococcus*, and *Pyxidicoccus* can enhance the efficiency of the mining of novel secondary metabolites through dereplication. However, the commonly used 16S rRNA gene sequencing cannot accurately differentiate members of the three genera above, and the whole-genome sequencing is unable to rapidly and inexpensively provide species assignation toward a large number of isolates. To overcome the limitations, the *gyrB* gene was investigated as a candidate genetic marker for exploring the phylogenetic relationships of bacteria within the three genera and for developing the *gyrB*-based typing method. Here, the bacterial phylogeny and species affiliations of the three genera were determined based on the phylogenomic reconstruction and the analysis of digital DNA–DNA hybridization values among 90 genomes, further confirming nine novel taxa and assigning over one-third of genomes to defined species. The phylogenetic relationships of these strains based on the *gyrB* gene sequences were congruent with those based on their genome sequences, allowing the use of the *gyrB* gene as a molecular marker. The *gyrB* gene-specific primers for the PCR-amplification and sequencing of bacteria within the three genera were designed and validated for 31 isolates from our group collection. The *gyrB*-based taxonomic tool proved to be able to differentiate closely related isolates at the species level. Based on the newly proposed 98.6% identity threshold for the 966-bp *gyrB* gene and the phylogenetic inference, these isolates were assigned into two known species and eight additional putative new species. In summary, this report demonstrated that the *gyrB* gene is a powerful phylogenetic marker for taxonomy and phylogeny of bacteria within the closely related genera *Myxococcus*, *Corallococcus*, and *Pyxidicoccus*, particularly in the case of hundreds or thousands of isolates in environmental studies.

## Introduction

The family *Myxococcaceae* belongs to the order *Myxococcales* and currently comprises five genera validly described as *Aggregicoccus*, *Corallococcus*, *Myxococcus*, *Pyxidicoccus*, and *Simulacricoccus*^1^. Just recently, the other four distinct genera *Archangium*, *Cystobacter*, *Hyalangium*, and *Stigmatella* have been proposed for reclassifying them into the family *Myxococcaceae* (Waite et al., 2020). Members of this family are widely distributed in terrestrial and marine environments (Liu et al., 2019), displaying great environmental adaptability. Among these genera above, the *Myxococcus*, *Corallococcus*, and *Pyxidicoccus* seem to be one of the most frequently isolated myxobacterial genera from almost all samples, especially from the soil (Dawid, 2000; Zhang et al., 2013; Wang et al., 2020) and cover the best studied and explored myxobacteria with a wide scope of research. For example, *Myxococcus xanthus* is most widely used as a model microorganism for studying bacterial social behaviors such as predation, swarming, fruiting-body formation, and sporulation (Zusman et al., 2007). The further fascinating feature of myxobacteria is that they can produce a large and variety of bioactive secondary metabolites acting as antimicrobials, antiparasitics, antivirals, cytotoxins, and anti-blood coagulants (Herrmann et al., 2017; Gregory et al., 2019). Many myxobacteria can prey on bacteria (Livingstone et al., 2017) and fungi (Li et al., 2019) by the secretion of antibiotic metabolites and hydrolytic enzymes, showing great application potential in plant disease control (Bull et al., 2002). Consequently, it is of great interest to be able to identify myxobacterial isolates, in a fast, reliable, and low-cost way, to determine their taxonomic affiliation, to monitor their ecological distribution and diversity in various environments.

The three genera *Myxococcus*, *Corallococcus*, and *Pyxidicoccus* are the most closely related among the family *Myxococcaceae*. From the early days to now, the morphology of cells and colonies and 16S rRNA gene analysis have been always used for the bacterial identification of the three genera above (Garcia et al., 2010; Reichenbach, 2015). However, the morphological characteristics such as typical shapes of vegetative cells and myxospores, the structure and color of fruiting bodies may vary under different culture conditions and media constituents and therefore are difficult to provide accurate myxobacterial taxonomic assignation (Reichenbach, 2015). These graphical and descriptive morphological features are also inconvenient for a data-based comparison among different research teams. Furthermore, the most frequently used 16S rRNA gene sequencing can only identify closely related strains at the genus level but not at the species level (Stackebrandt and Päuker, 2005; Stackebrandt et al., 2007). The multiple copies of the 16S rRNA gene in their genome may make the community abundance data distorted in microbiome surveys. With the advance of next-generation sequencing technologies, comparative genomics analysis based on the concatenated single-copy core genes has been successfully applied for identifying and classifying isolates of the three genera above (Chambers et al., 2020; Livingstone et al., 2020). For example, eight novel *Corallococcus* species, three novel *Myxococcus* species, and two novel *Pyxidicoccus* species have been recently proposed based on the analyses of comparative genomics and pan-genomics (Chambers et al., 2020; Livingstone et al., 2020), resulting in the number of described species to more than double. Although the genome-based analysis can produce reproducible and reliable phylogenetic relationships of bacteria within the three genera, it remains challenging according to the time-consuming and costly features, particularly in the case of hundreds or thousands of isolates. With the decrease in the sequencing cost and the advance in massive data analysis, it is reasonable to expect that the whole genome sequence-based comparison and characterization of bacteria within the three genera will become more feasible and practical than now. However, in the current case at least, the fast and accurate identification of bacteria belonged to the closely related genera *Myxococcus*, *Corallococcus*, and *Pyxidicoccus* is still a challenge whether using the 16S rRNA gene sequencing or whole-genome sequence comparison.

The conserved single-copy protein-coding genes, also known as housekeeping genes, have been proposed as alternative molecular markers for the study of microbial taxonomic relationships and diversity (Santos and Ochman, 2004; Vos et al., 2012; Ogier et al., 2019). Like the 16S rRNA gene, housekeeping genes are required for the maintenance of basic cellular functions and thus are essential and universally present in the bacterial kingdom. In contrast to the 16S rRNA gene, housekeeping genes are supposed to evolve at a much more rapid but constant rate and are therefore endowed with a better resolution power for differentiating different lineages that have recently diverged (Poirier et al., 2018). Moreover, single-copy housekeeping genes in bacterial genomes can avoid the overestimation of bacterial richness and abundance in ecological surveys based on 16S rRNA amplicon sequencing. As of now, some housekeeping genes have been used either to distinguish the closely related strains at the species level or to decipher microbial diversity (Poirier et al., 2018; Ogier et al., 2019). In the study of the heterogeneity of *Corallococcus coralloides* strains isolated from geographically diverse locations, the *gyrB* gene encoding DNA gyrase subunit B showed a higher resolution power against the 16S rRNA gene (Stackebrandt and Päuker, 2005). Soon after, the three housekeeping genes *csgA*, *fibA*, and *pilA* were used to investigate the small-scale genetic population structure of the soil bacterium *M. xanthus* (Vos and Velicer, 2006). The additional four taxonomic markers *lepA*, *fusA*, and *rpoB* were used to determine whether the genetic diversity of the three species within the genus *Corallococcus* is matched with their phenotypic properties (Stackebrandt et al., 2007). However, these molecular markers are too short to have enough genetic information to clearly distinguish the close relatives, and the use of these markers only focuses on a few species. More importantly, the classification criteria for these genes have not been established, to some extent due to the lack of myxobacterial genomic sequence at that time.

In recent years, with the rapid increase of myxobacterial isolates, it is urgent to establish an efficient method for the rapid and accurate identification of these bacteria. Many genome sequences belonged to the genera *Myxococcus*, *Corallococcus*, and *Pyxidicoccus* available in public databases provide an ideal opportunity for determining the threshold of a single molecular marker for species delineation. In comparison with other genetic markers, the *gyrB* gene with the much higher resolution has been fairly frequently used for determining phylogenetic relationships of closely related strains at the species level (Poirier et al., 2018; Martínez-Hidalgo et al., 2020; Klemetsen et al., 2021) and has been produced numerous reference sequences in public databases. Inspired by these results, we thus choose the *gyrB* gene as the candidate housekeeping gene. The present study aimed to determine the potential of the *gyrB* gene in the identification, genotyping, and phylogenetics of strains within the closely related three genera.

## Materials and Methods

### Determination of Genome Sequences Used in the Study

Genome sequences of bacteria within the three genera were retrieved from the NCBI database. The genomic quality was evaluated using the software CheckM version 1.1.2 (Parks et al., 2015). The general genomic characteristics of strains including genomic sizes and DNA G + C contents were conducted using the software QUAST version 5.0.2 (Gurevich et al., 2013). For consistency, all genome sequences were re-annotated using the software Prokka version 1.13 (Seemann, 2014). The 16S rRNA and *gyrB* gene sequences of each strain were extracted using a local BLAST search with default parameters. The digital DNA–DNA hybridization (dDDH) values were estimated using the genome-to-genome distance calculator (GGDC) version 2.1 online service with the recommended formula 2 (Auch et al., 2010). The dDDH values were visualized using the “HeatMap” tool of the software TBtools version 1.0981 (Chen et al., 2020). The pairwise identities of the 16S rRNA and *gyrB* gene sequences were conducted using the software DNAMAN version 8 (Lynnon Biosoft^2^) with default parameters after the multiple sequence alignment (MSA). The intraspecies and interspecies identities of the 16S rRNA and *gyrB* gene sequences were compared by the Student’s *t*-test using Microsoft Excel 2019. Correlation analyses between dDDH values, *gyrB*, and 16S rRNA gene sequences identities were undertaken by using the “basicTrendline” library in R^3^.

### Phylogenetic Analyses Based on the Genome, *gyrB*, and 16S rRNA Gene Sequences

To obtain core genes of genome sequences used in this study, the pan-genome analysis was performed using the software Bacterial Pan Genomes Analysis Pipeline (BPGA) version 1.3 with default parameters. The maximum-likelihood (ML) tree of the core genomes were reconstructed using the software IQ-TREE version 2.1.2 (Minh et al., 2020) based on the LG + F + R4 model of amino acid substitution with default parameters (Stamatakis, 2014), which was selected by the ModelFinder (Kalyaanamoorthy et al., 2017) according to the Bayesian information criterion (default) (Schwarz, 1978). For the *gyrB* and 16S rRNA gene sequences, the MSA was performed using the software MAFFT version 6.240 with the FFT-NS-2 algorithm (Katoh and Standley, 2013). And then, the ML trees based on the 16S rRNA and *gyrB* gene sequences were reconstructed by using the IQ-TREE, respectively, under the TIM2 + F + R2 and TIM + F + I + G4 nucleotide substitution models. Support for the two single-gene phylogenetic trees was inferred by ultrafast bootstrapping with 10,000 replicates (Hoang et al., 2018). The visualization and annotation of the resulting phylogenetic trees were performed using the software MEGA version X (Kumar et al., 2018). Strain *Aggregicoccus* sp. 17bor-14 was used as an outgroup in all phylogenetic analyses.

### The *gyrB* Gene Primer Design and Determination

To design primers of the *gyrB* gene for the amplification and sequencing of bacteria within the three genera, the complete *gyrB* gene sequences from genome sequences were aligned using the DNAMAN. A specific primer set of the *gyrB* gene was designed using the software Primer Premier version 5.0 (PREMIER Biosoft International, CA, United States). The 31 isolates that belonged to the above three genera based on the preliminary identification of the 16S rRNA gene sequencing (unpublished data) from our group were used for verifying the primer of the *gyrB* gene. All tested isolates were grown in the VY/2 medium (Reichenbach, 2015) at 28°C for a week. The genomic DNA of each isolate was extracted from fresh cells using the HiPure Bacterial DNA Kit (Magen Biotech Co., Ltd., Guangzhou, China) following the manufacturer’s instructions. The *gyrB* gene fragment was amplified in a 25 μL PCR mixture composed of 12.5 μL 2 × PCR Master Mix with 3 mmol/L MgCl_2_ (G-Clone Biotech Co., Ltd.), 0.5 μL each primer (10 mmol/L), 0.5 μL template DNA (ca. 50 ng/μL), and 11 μL deionized water. Gradient PCRs were performed to determine the optimal annealing temperature for the primers pair of the *gyrB* gene. The PCR reaction was done in a T100^TM^ Thermal Cycler (Bio-Rad, CA, United States) with the following thermal PCR profile: initial denaturation at 94°C for 5 min, 30 cycles of denaturation at 95°C for 30 s, annealing at 63°C for 30 s, and extension at 72°C for 70 s, followed by a final extension at 72°C for 10 min. PCR products were screened by electrophoresis on a 1% agarose gel and sequenced by Suzhou Genewiz Biotechnology Co., Ltd. (Suzhou, China). In this study, sequencing primers were the same as amplification primers. The genomic sequencing, *de novo* assembly, and quality assessment of representative isolates were determined based on the previously described methods (Liu et al., 2021). The 966-bp *gyrB* gene sequences identities and dDDH values between representative isolates and their closely related relatives were conducted by the DNAMAN and GGDC, respectively, as mentioned above.

### Nucleotide Sequence Accession Numbers

The sequence data generated in this study were deposited in the GenBank database under accession numbers provided in Supplementary Tables 1, 8.

## Results

### Selection and General Features of Genomes

**FIGURE 1:**
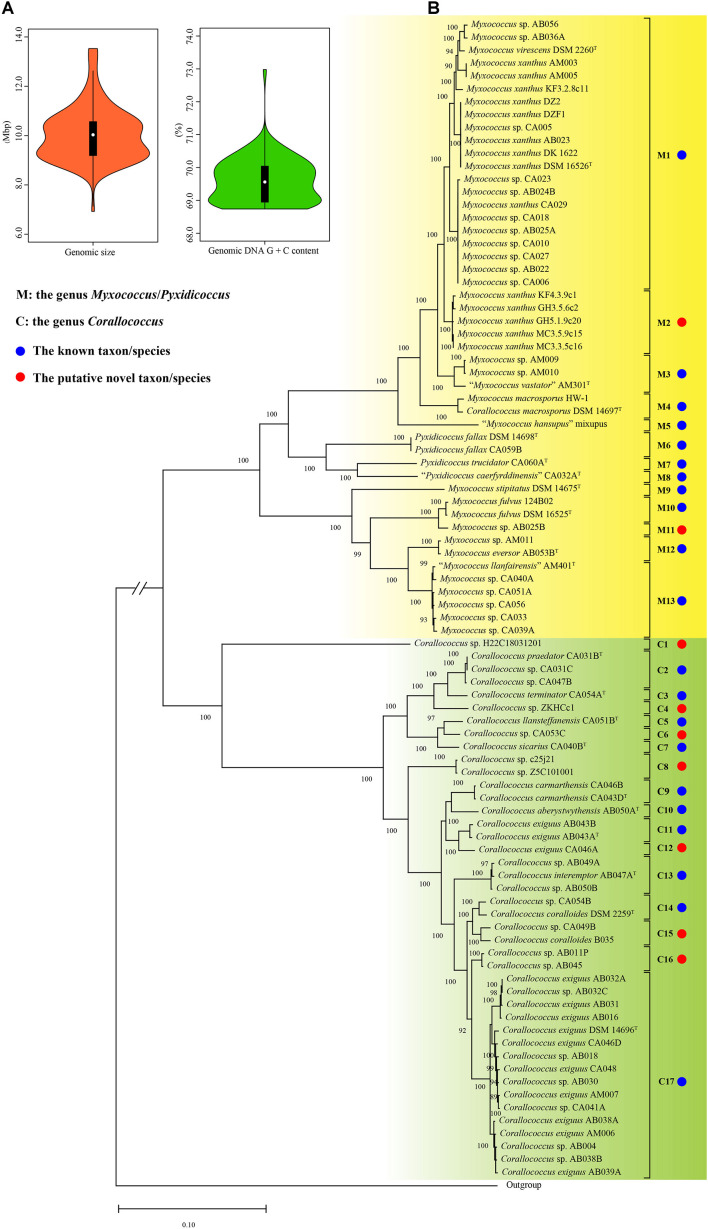
The violin plots of genomic sizes and DNA G + C contents **(A)**, the maximum-likelihood (ML) phylogenetic tree of 90 strains based on 1,887 orthologous protein sequences of 91 genome sequences **(B)**. The ML tree was reconstructed using the IQ-TREE with the LG + F + R4 model. The species names are effectively but not yet validly published and thus are in quotation marks. Strain *Aggregicoccus* sp. 17bor-14 was used as an outgroup. Bootstrap values great than 80% were shown at branch points. Bar: 0.1 represents the number of substitutions per site. The branch length of the outgroup was cut down to fit the image size and indicated by parallel oblique lines “//”.

A total of 106 genomes designated by the GenBank database as the genera *Myxococcus*, *Corallococcus*, and *Pyxidicoccus* were obtained in this study, also including that of strain *Aggregicoccus* sp. 17bor-14 as an outgroup. To ensure the reliability of the subsequent analysis, the quality of all genomes was assessed using the CheckM. Based on the estimation from the CheckM, 94 genomes were to be considered high quality based on more than 95% of completeness and less than 5% of contamination. Meanwhile, for multiple genomes of the same strain with different numbers, only one genome with the highest quality was selected for analysis. Therefore, there were 14 complete and 77 draft genomes used in this study (Table 1). The general features of genomes are summarized in Table 1. The genomic sizes ranged from 8.80 Mbp (*Myxococcus* sp. AM009) to 13.53 Mbp (*Pyxidicoccus fallax* DSM 14698^T^) with an average of 10.10 ± 1.07 Mbp. The genomic DNA G + C contents ranged from 68.74% (“*Myxococcus llanfairensis*” AM401^T^, this name originated from “*Myxococcus* llanfairpwllgwyngyllgogerychwyrndrobwllllantysiliogogogochensis” and was abbreviated to “*M. llanfairensis*”, the same below) to 70.74% (*Corallococcus* sp. Z5C101001) with a mean of 69.57 ± 0.59%. These results indicated that bacteria of the three genera above had two distinctive features with large genomes and high genomic DNA G + C contents relative to most bacterial taxa (Figure 1A) (Whitworth and Zwarycz, 2020).

**TABLE 1:** The detailed information on genomes used in this study.

Original name	Group	Species name	Accession number	Size (Mbp)	Genomic DNA G + C content (%)	Complete ness (%)	Contamin ation (%)
*Myxococcus* sp. AB056	M1	*M. virescens*/*xanthus*	VHLB00000000	9.11	69.07	98.7	0.8
*Myxococcus* sp. AB036A	M1	*M. virescens*/*xanthus*	VHLC00000000	9.27	69.01	99.4	2.0
*Myxococcus virescens* DSM 2260^T^	M1	*M. virescens*/*xanthus*	FNAJ00000000	9.24	69.18	99.4	1.3
*Myxococcus xanthus* AM003	M1	*M. virescens*/*xanthus*	JABFNS00000000	9.14	69.22	98.7	1.3
*Myxococcus xanthus* AM005	M1	*M. virescens*/*xanthus*	JABFNT00000000	9.15	69.22	98.7	2.6
*Myxococcus xanthus* KF3.2.8c11	M1	*M. virescens*/*xanthus*	CP017171	8.95	68.99	100	0
*Myxococcus xanthus* DZ2	M1	*M. virescens*/*xanthus*	AKYI00000000	9.27	68.89	99.4	1.3
*Myxococcus xanthus* DZF1	M1	*M. virescens*/*xanthus*	AOBT00000000	9.28	68.89	99.4	1.3
*Myxococcus* sp. CA005	M1	*M. virescens*/*xanthus*	SRLV00000000	9.11	68.91	98.7	1.3
*Myxococcus xanthus* AB023	M1	*M. virescens*/*xanthus*	JABFNQ00000000	9.13	68.91	98.7	1.3
*Myxococcus xanthus* DK 1622	M1	*M. virescens*/*xanthus*	CP000113	9.14	68.89	100	0
*Myxococcus xanthus* DSM 16526^T^	M1	*M. virescens*/*xanthus*	FNOH00000000	9.26	68.89	99.4	1.3
*Myxococcus* sp. CA023	M1	*M. virescens*/*xanthus*	JAAEAH00000000	9.08	68.89	98.7	0.7
*Myxococcus* sp. AB024B	M1	*M. virescens*/*xanthus*	SRLY00000000	9.06	68.88	98.7	0.7
*Myxococcus xanthus* CA029	M1	*M. virescens*/*xanthus*	JABFNR00000000	9.19	68.87	98.7	3.8
*Myxococcus* sp. CA018	M1	*M. virescens*/*xanthus*	JAAEAG00000000	9.07	68.86	98.7	1.3
*Myxococcus* sp. AB025A	M1	*M. virescens*/*xanthus*	SRLX00000000	9.05	68.88	98.7	0.7
*Myxococcus* sp. CA010	M1	*M. virescens*/*xanthus*	VHLA00000000	9.05	68.89	98.4	0.7
*Myxococcus* sp. CA027	M1	*M. virescens*/*xanthus*	WBSK00000000	9.05	68.88	98.7	0.7
*Myxococcus* sp. AB022	M1	*M. virescens*/*xanthus*	VHLD00000000	9.06	68.89	98.7	0.8
*Myxococcus* sp. CA006	M1	*M. virescens*/*xanthus*	SRLU00000000	9.05	68.88	98.7	0.7
*Myxococcus xanthus* KF4.3.9c1	M2	MPNS1^#^	CP017172	9.43	68.92	100	0
*Myxococcus xanthus* GH3.5.6c2	M2	MPNS1^#^	CP017169	9.32	69.01	100	0
*Myxococcus xanthus* GH5.1.9c20	M2	MPNS1^#^	CP017170	9.26	68.98	100	0
*Myxococcus xanthus* MC3.5.9c15	M2	MPNS1^#^	CP017174	9.32	68.97	100	0
*Myxococcus xanthus* MC3.3.5c16	M2	MPNS1^#^	CP017173	9.32	68.97	100	0
*Myxococcus* sp. AM009	M3	“*M. vastator*”	JABXEP00000000	8.80	70.07	98.7	2.6
*Myxococcus* sp. AM010	M3	“*M. vastator*”	JABXEO00000000	8.93	70.05	98.7	1.3
“*Myxococcus vastator*” AM301^T^	M3	“*M. vastator*”	JAAIYB00000000	8.99	69.92	98.1	2.6
*Myxococcus macrosporus* HW-1	M4	*M. macrosporus*	CP002830	9.00	70.63	100	0
*Corallococcus macrosporus* DSM 14697^T^	M4	*M. macrosporus*	CP022203	8.97	70.62	100	0
“*Myxococcus hansupus*” mixupus	M5	“*M. hansupus*”	CP012109	9.49	69.17	100	0
*Pyxidicoccus fallax* DSM 14698^T^	M6	*P*. *fallax*	JABBJJ00000000	13.53	70.48	99.7	3.9
*Pyxidicoccus fallax* CA059B	M6	*P*. *fallax*	JABJTR00000000	13.39	70.45	99.0	3.9
*Pyxidicoccus trucidator* CA060A^T^	M7	*P*. *trucidator*	JAAIXZ00000000	12.67	70.30	100.0	2.1
“*Pyxidicoccus caerfyrddinensis*” CA032A^T^	M8	“*P*. *caerfyrddinensis*”	JAAIYA00000000	13.43	70.21	100.0	2.6
*Myxococcus stipitatus* DSM 14675^T^	M9	*M*. *stipitatus*	CP004025	10.35	69.19	100	0
*Myxococcus fulvus* 124B02	M10	*M*. *fulvus*	CP006003	11.05	69.96	100	0
*Myxococcus fulvus* DSM 16525^T^	M10	*M*. *fulvus*	FOIB00000000	10.82	70.00	99.4	0.7
*Myxococcus* sp. AB025B	M11	MPNS2^#^	SRLW00000000	10.60	70.15	98.6	0.7
*Myxococcus* sp. AM011	M12	*M*. *eversor*	JABXEM00000000	11.62	68.87	98.7	1.6
*Myxococcus eversor* AB053B^T^	M12	*M*. *eversor*	JAAIXY00000000	11.39	68.93	99.4	2.0
“*Myxococcus llanfairensis*” AM401^T^	M13	“*M*. *llanfairensis*”	VIFM00000000	12.41	68.74	99.4	4.3
*Myxococcus* sp. CA040A	M13	“*M*. *llanfairensis*”	JABUMR00000000	11.72	68.94	99.4	1.3
*Myxococcus* sp. CA051A	M13	“*M*. *llanfairensis*”	JABUMS00000000	11.45	68.90	99.4	1.3
*Myxococcus* sp. CA056	M13	“*M*. *llanfairensis*”	JABUMT00000000	11.36	68.93	99.4	2.0
*Myxococcus* sp. CA033	M13	“*M*. *llanfairensis*”	JABUMU00000000	11.62	68.85	99.4	1.3
*Myxococcus* sp. CA039A	M13	“*M*. *llanfairensis*”	JABUMQ000000000	11.59	68.78	98.7	1.7
*Corallococcus* sp. H22C18031201	C1	CPNS1*	QNUN00000000	9.07	69.51	99.4	0.8
*Corallococcus praedator* CA031B^T^	C2	*C*. *praedator*	RAWI00000000	10.51	69.89	98.7	5.1
*Corallococcus* sp. CA031C	C2	*C*. *praedator*	RAWH00000000	10.23	69.91	98.7	1.3
*Corallococcus* sp. CA047B	C2	*C*. *praedator*	RAWD00000000	10.34	69.92	98.7	1.3
*Corallococcus terminator* CA054A^T^	C3	*C*. *terminator*	RAVZ00000000	10.35	69.56	98.7	2.0
*Corallococcus* sp. ZKHCc1	C4	CPNS2*	JAAIYO00000000	9.44	70.64	98.7	0.7
*Corallococcus llansteffanensis* CA051B^T^	C5	*C*. *llansteffanensis*	RAWB00000000	10.53	70.35	98.7	3.2
*Corallococcus* sp. CA053C	C6	CPNS3*	RAWA00000000	10.50	70.18	98.7	4.6
*Corallococcus sicarius* CA040B^T^	C7	*C*. *sicarius*	RAWG00000000	10.39	70.25	99.4	1.3
*Corallococcus* sp. c25j21	C8	CPNS4*	JAAAPJ00000000	9.23	70.69	99.4	0.1
*Corallococcus* sp. Z5C101001	C8	CPNS4*	VKLU00000000	9.08	70.74	98.7	0.1
*Corallococcus carmarthensis* CA046B	C9	*C*. *carmarthensis*	JABFJX00000000	10.74	69.91	99.4	3.3
*Corallococcus carmarthensis* CA043D^T^	C9	*C*. *carmarthensis*	RAWE00000000	10.79	69.94	99.4	2.4
*Corallococcus aberystwythensis* AB050A^T^	C10	*C*. *aberystwythensis*	RAWK00000000	9.98	70.01	98.7	1.4
*Corallococcus exercitus* AB043B	C11	*C*. *exercitus*	JABFJV00000000	10.26	70.26	99.4	2.4
*Corallococcus exercitus* AB043A^T^	C11	*C*. *exercitus*	RAVW00000000	10.15	70.32	99.4	1.4
*Corallococcus exercitus* CA046A	C12	CPNS5*	JABFJW00000000	9.90	70.55	98.7	0.4
*Corallococcus* sp. AB049A	C13	*C*. *interemptor*	RAWL00000000	9.51	70.09	98.7	4.2
*Corallococcus interemptor* AB047A^T^	C13	*C*. *interemptor*	RAWM00000000	9.47	70.11	99.4	0.8
*Corallococcus* sp. AB050B	C13	*C. interemptor*	RAWJ00000000	9.40	70.12	98.7	0.8
*Corallococcus* sp. CA054B	C14	*C*. *coralloides*	RAVY00000000	9.91	69.98	99.4	0.8
*Corallococcus coralloides* DSM 2259^T^	C14	*C*. *coralloides*	CP003389	10.08	69.90	100	0
*Corallococcus* sp. CA049B	C15	CPNS6*	RAWC00000000	9.63	70.23	98.7	0.7
*Corallococcus coralloides* B035	C15	CPNS6*	CP034669	9.59	70.26	100	0
*Corallococcus* sp. AB011P	C16	CPNS7*	RAVX00000000	10.18	69.79	99.4	0.8
*Corallococcus* sp. AB045	C16	CPNS7*	RAWN00000000	9.94	69.87	98.7	0.8
*Corallococcus exiguus* AB032A	C17	*C*. *exiguus*	JABJTS00000000	10.44	69.62	98.7	3.3
*Corallococcus* sp. AB032C	C17	*C*. *exiguus*	RAWP00000000	10.45	69.55	99.4	1.3
*Corallococcus exiguus* AB031	C17	*C*. *exiguus*	JABEKZ00000000	10.43	69.72	98.7	1.0
*Corallococcus exiguus* AB016	C17	*C*. *exiguus*	JABEKY00000000	10.75	69.56	98.7	3.0
*Corallococcus exiguus* DSM 14696^T^	C17	*C*. *exiguus*	JAAAPK00000000	10.41	69.60	99.4	1.3
*Corallococcus exiguus* CA046D	C17	*C*. *exiguus*	JABNNE00000000	10.50	69.61	98.7	3.7
*Corallococcus* sp. AB018	C17	*C*. *exiguus*	RAWR00000000	10.45	69.55	99.4	1.3
*Corallococcus exiguus* CA048	C17	*C*. *exiguus*	JABELB00000000	10.35	69.61	98.7	2.2
*Corallococcus* sp. AB030	C17	*C*. *exiguus*	RAWQ00000000	10.64	69.61	98.7	2.0
*Corallococcus exiguus* AM007	C17	*C*. *exiguus*	JABNNG00000000	10.46	69.59	99.4	1.3
*Corallococcus* sp. CA041A	C17	*C*. *exiguus*	RAWF00000000	10.26	69.60	99.4	3.0
*Corallococcus exiguus* AB038A	C17	*C*. *exiguus*	JABJTT00000000	10.57	69.48	98.7	2.0
*Corallococcus exiguus* AM006	C17	*C*. *exiguus*	JABNNF00000000	10.59	69.51	99.4	3.5
*Corallococcus* sp. AB004	C17	*C*. *exiguus*	RAWS00000000	10.60	69.47	98.7	2.2
*Corallococcus* sp. AB038B	C17	*C*. *exiguus*	RAWO00000000	10.77	69.45	99.4	0.7
*Corallococcus exiguus* AB039A	C17	*C*. *exiguus*	JABJTU00000000	10.54	69.47	99.4	0.7
*Aggregicoccus* sp. 17bor-14	–	–	VJZZ00000000	6.93	72.98	98.7	1.3

^#^MPNS, *Myxococcus* putative new species. *CPNS, *Corallococcus* putative new species. The species names are effectively but not yet validly published and thus are in quotation marks. Genomic sizes and DNA G + C contents were determined using the QUAST. The genomic completeness and contamination were assessed using the CheckM.

### Phylogeny and Species Delineation Based on Genome Sequences

A total of 1887 orthologous protein sequences (Supplementary Table 2) from the core genome by the BPGA were used to infer the phylogeny of 90 bacteria within the three genera. As shown in Figure 1B, the ML phylogenomic tree was characterized by high bootstrap values, indicating that the protein sequences selected were reflective of a robust evolutionary relatedness between bacteria. The ML tree showed that bacteria of the three genera were grouped into two distinct groups. Group M was composed of 48 strains, including eleven type strains and 37 non-type strains. In Group M, the bacteria from the two genera *Myxococcus* and *Pyxidicoccus* were mixed. Similar to the study by Chambers et al. (2020), the two genera were therefore proposed as a single genus, referring to it hereafter as the genus *Myxococcus*/*Pyxidicoccus*. Group C was found to be comprised of ten type strains and 32 non-type strains and matched exactly to the genus *Corallococcus*. In the phylogenomic tree, the type strain *Corallococcus macrosporus* DSM 14697^T^ was located in Group M and shared a close relationship with strain *Myxococcus macrosporus* HW-1. Actually, these studies have proved that strain *C. macrosporus* DSM 14697^T^ was more closely related to members of the genus *Myxococcus* than to those of the genus *Corallococcus* (Lang and Stackebrandt, 2009). But, *M. macrosporus* is still shown to be a homotypic synonym of *C. macrosporus* in the LPSN^4^. From a taxonomic standpoint, it is necessary to clarify in the following study.

The DNA–DNA hybridization (DDH) values have been used continuously for over half a century as the gold standard for prokaryotic species circumscription at the genomic level (Chun et al., 2018) and can be obtained from the GGDC. In contrast with DDH values, various algorithms available for calculating the average nucleotide identity could produce different values and thus did not provide consistent results regarding the conspecificity of isolates (Palmer et al., 2020). In view of this situation, in the current study, the species assignation for all strains was determined only based on the dDDH and phylogenomic analyses and was also used as a criterion in the following analyses of the *gyrB* and 16S rRNA genes. Based on the 70% DDH threshold, all strains were divided into 30 species (Supplementary Figure 1 and Supplementary Table 3). Group M included 13 species (marked with M1 to M13) corresponding to 11 well-defined species indicated by blue solid circles (the same below) and two putative novel species indicated by red solid circles (the same below). Group C contained 17 species (marked with C1–C17) corresponding to 10 known species and seven putative novel species. Based on the new species assignation, more than one-third of genomes with unresolved or incorrect specific epithets were reclassified into defined species in this study (Table 1). Unexpectedly, the two type strains *Myxococcus virescens* DSM 2260^T^ and *M. xanthus* DSM 16526^T^ both belonged to the M1 taxon and shared a 73.1% dDDH above the threshold for bacterial species delineation, indicating that they should be conspecific. In this case, the M1 taxon was preliminarily designated as the species *M. virescens*/*xanthus* in this study, as shown in Table 1. As a result, combining with the phylogenomic and dDDH values analyses, a robust phylogeny of the genera *Myxococcus*, *Corallococcus*, and *Pyxidicoccus* and reliable species assignation of their bacteria were conducted and would provide a solid foundation for establishing a new single housekeeping gene-based identification of bacteria within the three genera.

### Phylogeny of *gyrB* and 16S rRNA Gene Sequences

**FIGURE 2:**
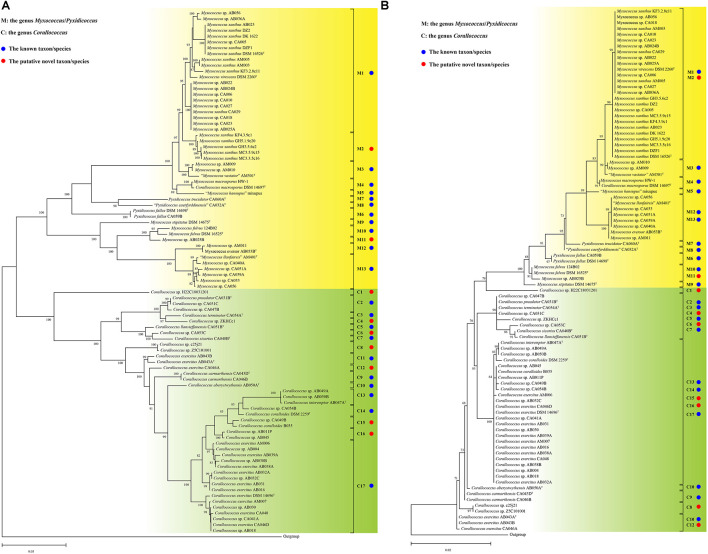
The ML phylogenetic trees based on complete *gyrB* **(A)** and 16S rRNA gene **(B)** sequences. The ML trees of the *gyrB* and 16S rRNA gene were reconstructed using the IQ-TREE with the TIM + F + I + G4 and TIM2 + F + R2 models, respectively. The species names are effectively but not yet validly published and thus are in quotation marks. Strain *Aggregicoccus* sp. 17bor-14 was used as an outgroup. Bootstrap values great than 80% in *gyrB*-based tree and 50% in 16S rRNA-based tree were shown at branch points. Bar: 0.05/0.02 represents the number of substitutions per site.

Inspired by previous studies (Stackebrandt and Päuker, 2005; Stackebrandt et al., 2007), the *gyrB* gene was used as a candidate marker to infer the bacterial phylogeny of the genera *Myxococcus*, *Corallococcus*, and *Pyxidicoccus* in this study. For all 90 strains analyzed, only one copy of the *gyrB* gene was identified in each genome sequence. The complete *gyrB* gene sequences varied in size between 2,448 and 2,466 bp. As shown in Figure 2A, similar to the genome-based tree, all strains in the *gyrB* gene-based tree were also clustered into two distinct groups. At the species level, all strains in the *gyrB* gene-based tree were further divided into 30 subgroups, matching exactly 30 species from the phylogenomic and dDDH values analyses. Some small differences in tree topologies of the genome- and *gyrB*-based trees were observed. For example, the two species C9 and C10 were found to be sister taxa in the genome-based tree, while the species C9 was the outer taxon against the species C10 in the *gyrB* gene-based tree. A similar situation was found for the two species C11 and C12. In more detail, the intraspecific phylogenetic relationships of some strains in the *gyrB* gene-based tree were different from those in the genome-based tree (Figures 1B, 2A). The intraspecies identities of the *gyrB* gene sequences ranged from 97.3 to 100% with a mean value of 98.8%, and the interspecies identities ranged from 85.7 to 98.5% with a mean value of 89.5% (Supplementary Figure 2 and Supplementary Table 4). The intraspecies identities of the complete *gyrB* gene sequences were significantly higher than and interspecies identities (*t*-test, *p* < 0.001, Supplementary Figure 2). These results indicated that the *gyrB* gene can provide reliable evolutionary relationships and species assignation of the three genera and thus seems to be a better alternative as a powerful molecular marker to infer their phylogeny.

The lengths of complete 16S rRNA gene sequences from the respective genome ranged from 1,536 to 1,538 bp. Among all genomes, each complete genome contained the same three or four copies of the 16S rRNA gene and each high-quality draft genome contained only one copy (data not shown). Therefore, a complete 16S rRNA gene sequence in each genome was obtained for the phylogenetic analysis. In the phylogenetic tree based on 16S rRNA gene sequences, many species that were well differentiated based on genome and *gyrB* gene sequences were clustered together (Figure 2B). For example, the two species M1 and M2 were on the same branch. Similar situations were observed for several other species, such as species M10–M11, M12–M13, C2–C7, and C13–C17. The phylogenetic relationships of 90 strains in the 16S rRNA gene-based tree were significantly different from those in the genome- and *gyrB* gene-based trees. One of the striking differences was that the five species C13–C17 clustered together in 16S rRNA gene tree, but they formed five different clusters/species in the genome- and *gyrB* gene-based trees. More obviously, the phylogenetic tree of 16S rRNA gene sequences presented much shorter branch lengths and lower bootstrap values than those of genome- and *gyrB*-based trees. As shown in Supplementary Figure 2, the intraspecies identities of 16S rRNA gene sequences ranged from 99.7 to 100% with a mean value of 99.97%, and the interspecies identities ranged from 97.2 to 100% with a mean value of 98.4% (Supplementary Table 5). The intraspecies identities of the 16S rRNA gene sequences were significantly higher than the interspecies identities (*t*-test, *p* < 0.001, Supplementary Figure 2). The same significance levels of identities of the *gyrB* and 16S rRNA gene sequences did not match their distinct phylogenetics. The inconsistency was mainly due to over-representative identities between strains of some subgroups capable of being distinguished by the 16S rRNA gene. In short, the 16S rRNA gene was inappropriate for accurate identification of bacteria within of the closely related three genera due to its poor resolution.

### Establishment of the *gyrB* Gene-Based PCR Method

The above-described *in silico* analyses demonstrated that the *gyrB* gene is a powerful phylogenetic marker to differentiate bacteria of the closely related three genera at the species level. Therefore, a PCR-based method that could be widely used by researchers working with the three genera would be extremely valuable as a simple tool in the classification of isolates.

**FIGURE 3:**
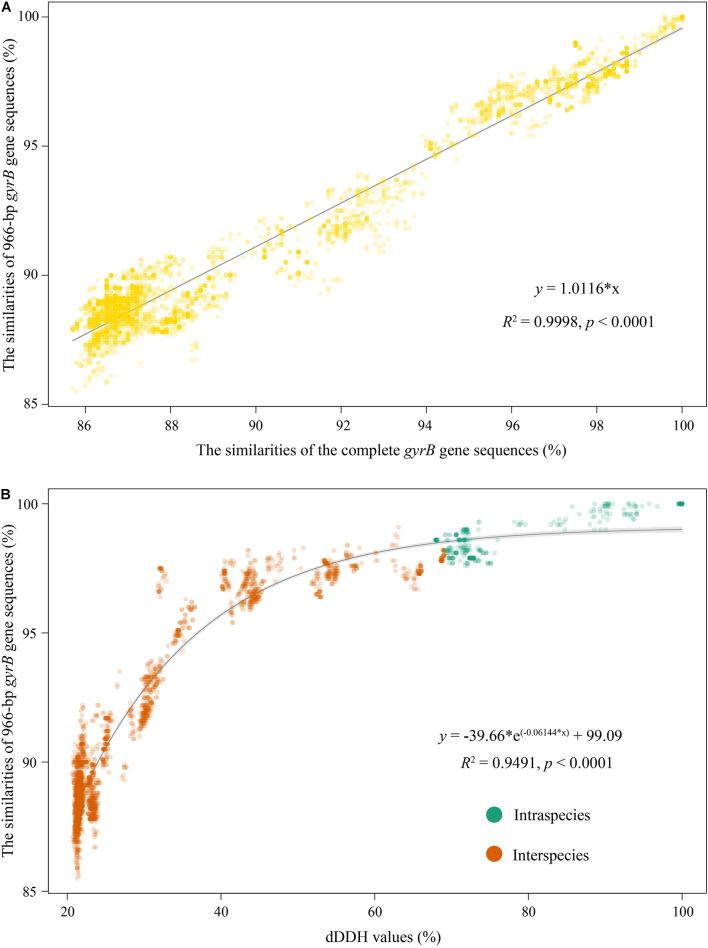
Correlation analyses of identities between the complete *gyrB* gene sequences and 966-bp *gyrB* ones **(A)**, and the dDDH values and the 966-bp *gyrB* gene sequence identities **(B)**. Correlation analyses of the former and latter were, respectively, simulated using the Excel linear regression and “exp3P” model of the “basicTrendline” library in R.

To establish the *gyrB* gene-based PCR method, amplification primers of this gene were first designed based on 90 complete *gyrB* gene sequences from their genomes. The pair of primers covering a 1,079 bp fragment without any insertions and deletions (corresponding to the position 348–1,426 within the complete *gyrB* gene sequence from type strain *Myxococcus fulvus* DSM 16525^T^ with the locus_tag = “SAMN05443572_101133”) was proposed: Myxoco_gyrBF (5′-AGCAAGTTCGGCAACG G-3′) and Myxoco_gyrBR (5′-AGCATCTTCTCGAAGCG-3′). Temperature gradients from 56 to 66°C were performed for confirming an optimum annealing temperature in PCR. The optimal annealing temperature was chosen at 63°C based on the detection of the brightness and uniqueness of PCR products/brands in agarose gel electrophoresis (Supplementary Figure 3). Logically, strains isolated from different habitats usually have more genotypes, and the use of more genotypic myxobacteria is conducive to better validate this method. Therefore, in this study, 31 bacteria isolated from multiple sources (Supplementary Table 1) by our group were related to the three genera *Myxococcus*, *Corallococcus*, and *Pyxidicoccus* based on the preliminary 16S rRNA gene sequencing analysis (unpublished data) and were used as target strains. All tested strains produced the expected PCR fragments of the *gyrB* gene and PCR products were sequenced using the amplification primers. After the MSA of the *gyrB* gene, all ambiguous bases at both ends were trimmed and the final sequence length for comparison was 966 bp. Phylogenetic tree based on 966-bp *gyrB* gene sequences from 31 tested isolates and 90 strains were reconstructed, as shown in Supplementary Figure 4. Among all tested isolates, 16 were distributed between the above four subgroups M1, M11, C8, and C9; 15 formed eight independent subgroups different from the above 30 ones obtained by the genome analysis. The comparison of the 966-bp *gyrB* gene sequences from 90 genomes showed that the intraspecies identities ranged from 97.6 to 100% with a mean value of 98.5%, and the interspecies identities ranged from 88.5 to 99.1% with a mean value of 90.7% (Supplementary Figure 2 and Supplementary Table 6). And apparently, an overlapped area between intraspecies and interspecies identities was from 97.6 to 99.1% (Supplementary Figure 2). Interestingly, almost all identities in the overlapping area came from the two subgroups M1 and C17 (Supplementary Tables 6, 7). But, as shown in Supplementary Figure 4, the accurate identification of these strains that were in the identity overlapped area was resolved using the phylogenetic analysis of the 966-bp *gyrB* gene. For example, strains AB036A and DZF1 shared a 97.7% identity in the overlapping area, but they could cluster into the subgroup M1. The identities of the complete *gyrB* gene sequences and the 966-bp ones were highly linear (*R*^2^ = 0.9998, Figure 3A), suggesting that their ability to infer phylogenetic relationships of bacteria within the three genera was almost equal. In addition, the intraspecies identities of the 966-bp *gyrB* gene sequences were statistically significantly higher than the interspecies identities (*t*-test, *p* < 0.001, Supplementary Figure 2). These results indicated that the PCR primers designed in this study were suitable for amplifying and sequencing the target region of the *gyrB* gene for bacteria of the three genera.

### Determination of the *gyrB* Gene Threshold for Species Delineation

The threshold of the *gyrB* gene for species delineation is an important parameter in the identification of strains with the genera *Myxococcus*, *Corallococcus*, and *Pyxidicoccus*. The correlation between dDDH values and marker gene sequence identities shows the accuracy with which a marker gene reflects the genome variation rate and indicates how precisely a marker gene predicts the phylogenetic relationship of genome sequences between strains. Consequently, we attempted to determine the identity threshold of the *gyrB* gene for species delineation using the correlation analysis. The dDDH values were highly correlated with identities of the complete *gyrB* gene sequences (*R*^2^ = 0.9760 and *p* < 0.0001, Supplementary Figure 5) and the 966-bp ones (*R*^2^ = 0.9491 and *p* < 0.0001, Figure 3B). This indicated that the change rate in nucleotide sequences of the complete *gyrB* gene was consistent with that of the 966-bp ones for bacteria of the three genera. Based on respective regression equations, 70% dDDH was equivalent to 98.4% of the complete *gyrB* gene or 98.6% of the 966-bp *gyrB* gene, both of which were used as thresholds for species delineation of bacteria within the three genera.

Applying the 98.6% threshold of the 966-bp *gyrB* gene, 16 isolates were assigned into the four known species M1, M11, C8, and C9, while the other 15 isolates were divided into the eight putative novel species. To further confirm these new species, the genomic sequences of the five representative isolates were determined in this study. Based on the estimation from the CheckM, the five genome sequences were also of high quality by comparing the standard above (Supplementary Table 8). The *gyrB* gene sequences of the five isolates extracted from each genome using a local BLAST search were the same as those obtained by the PCR, further confirming the authenticity of the final genome assembly. As shown in Supplementary Table 9, the dDDH values between strains AB025B, XM-1-1-1, and AS-1-15 were above the 70% threshold for bacterial species delineation, while those between each strain from the species M11 and other strains were below this threshold, indicating that M11 should represent a novel genospecies. The dDDH value between isolates AS-1-6 and AS-1-12 was 90.2%, demonstrating that they should belong to the same genospecies. The dDDH values between each isolate (AS-1-6 and AS-1-12) and other strains, RHSTA-1-4 and other strains were below the 70% threshold. The dDDH values analysis of these five representative isolates strengthened the accuracy of species assignation based on the 966-bp *gyrB* gene analysis. As a result, the 98.6% identity of the 966-bp *gyrB* gene can be used as a reliable threshold for rapidly and accurately identifying isolates of the above three most closely related genera to the species level in practice. In addition, for these potential new taxa, we will endeavor to determine phenotypic and genotypic characteristics to describe them as novel species in the future.

## Discussion

The current phylogenomic study demonstrated that the *gyrB* gene sequencing is a powerful molecular tool for identifying and classifying bacteria of the closely related genera *Myxococcus*, *Corallococcus*, and *Pyxidicoccus* at the species level. The analyses of the complete and 966-bp *gyrB* gene sequences showed that this marker provides accurate phylogenetic relationships of these bacteria consistent with genomic analysis and has greater discriminatory power than the widely used 16S rRNA gene, in particular when analyzing closely related strains.

The 16S rRNA gene has been a mainstay for the classification and identification of members of myxobacteria for decades (Shimkets and Woese, 1992; Garcia et al., 2010; Mohr, 2018). However, the 16S rRNA gene (which represents only 0.03% of an average 5-Mbp prokaryotic genome) shows a limited resolution for highly related strains at the interspecies and even intergeneric levels, making reliable species and genus level identifications not possible. In the last decade, with the advance of whole-genome sequencing and bioinformatic tool development, many genome-based methods have been developed and applied for microbial taxonomy, for example, the pan-genome analysis (Chambers et al., 2020; Livingstone et al., 2020) and the dDDH analysis (Auch et al., 2010), both of which have also been used to distinguish closely related bacteria in this study. However, considering that the whole-genome sequencing and analysis for a large number of isolates was a costly, time- and computer resource-consuming process and required bioinformatic skills for data processing and the effective interpretation of results, comparison of the DNA sequences of protein-encoding genes is an alternative approach to the analysis of whole-genome relatedness. Therefore, multiple housekeeping genes have been used as group-level taxonomic markers for some myxobacterial taxa (Stackebrandt and Päuker, 2005; Stackebrandt et al., 2007). But the use of these marker genes was only used for one or several species of the genera *Myxococcus* and *Corallococcus*, and was also a lack of group- or species-specific primers, resulting in negative amplification reactions for some isolates. In this study, a simple and accurate *gyrB* gene-based PCR method extending to the three genera and with specific primers was proposed and can bridge the gap between 16S rRNA gene sequence and genome analyses for taxonomic affiliation and phylogenetic relationships of bacteria within the three closely related genera above.

Species are regarded as the fundamental units in the taxonomy of bacteria and archaea, and the standard of species demarcation is an important parameter for taxonomic studies (Rosselló-Móra and Amann, 2015). For the myxobacteria, the assignation of strains to known or novel species is often performed using the morphology of vegetative cells, swarming colonies, fruiting bodies, and myxospores (Garcia and Müller, 2014). But, due to the lack of clear and quantifiable standards for species definition and over-reliance on personal experience and skills, these morphological characteristics seem somewhat subjective and unsuitable as a practical taxonomic criterion. Therefore, single-locus nucleotide-based and genome-based approaches have been applied for the myxobacterial taxonomy, such as the 16S rRNA gene (Chambers et al., 2020), a single housekeeping gene (Stackebrandt et al., 2007), and core genes (Livingstone et al., 2020). Among these approaches, the traditional 97% (Stackebrandt and Goebel, 1994) or updated 98.65% (Kim et al., 2014) of 16S rRNA gene sequence identities are not suitable as the thresholds for species delineation of highly related strains such as those from the genera *Myxococcus*, *Corallococcus*, and *Pyxidicoccus*, and previous studies did not provide thresholds of these molecular markers for species delineation (Stackebrandt and Päuker, 2005; Stackebrandt et al., 2007). To address the issue, the threshold for species delineation of the three genera in the current study was proposed through phylogenomics and the correlation analysis of dDDH values and the *gyrB* gene identities, and will greatly improve its practicability and comparability. The use of the *gyrB* gene threshold for the accurate and rapid identification of large batches of environmental isolates at the species level is advantageous in terms of resolution but also from a practical and financial point of view. Moreover, with the dramatic increase of the *gyrB* gene sequences in public databases, we anticipate that the *gyrB* gene will also become an interesting target to characterize the *in situ* diversity and abundance of the three genera and to guide further isolation endeavors from environmental samples.

Myxobacteria represents a highly diverse and ubiquitous group. As the most frequently isolated myxobacteria, the accurate, affordable, and fast identification of bacteria within the three genera *Myxococcus*, *Corallococcus*, and *Pyxidicoccus* is a major challenge owing to the limited discriminative power of the 16S rRNA gene. The current study has proposed a useful molecular identification tool using the *gyrB* gene sequencing as an alternative to the traditional 16S rRNA gene. Following this study, some potential important topics, but not limited to these, will be interesting to be determined by us and/or other researchers: (i) improving and then extending the *gyrB*-based method for strain identification to other taxa or even the whole myxobacteria, rather than just concentrating on the genera *Myxococcus*, *Corallococcus*, and *Pyxidicoccus*; (ii) establishing a curated DNA sequence database and a web-based tool for *gyrB*-based identification of myxobacterial isolates for much broader and easier use of this genetic marker in worldwide researchers; (iii) developing a *gyrB* amplicon sequencing protocol compatible with the high-throughput sequencing platform for profiling *in situ* myxobacterial community structure, species diversity, and temporal distribution from environmental samples; (iv) clarifying the taxonomic status of the genus *Pyxidicoccus* and the species *C. macrosporus* and identifying the potential new taxa obtained from this study using the polyphasic taxonomic approach in the subsequent study.

## Conclusion

In this report, we have contributed the framework of phylogenetic relationships of bacteria within the genera *Myxococcus*, *Corallococcus*, and *Pyxidicoccus*, and determines the well-suited *gyrB* gene for species assignation. We believe that the discovery of the phylogenetic power of the *gyrB* gene and the establishment of a PCR method that can be used in amplification and sequencing of the gene is of general interest, whether for use alone or together with the genome-based analysis. This research also provides a paradigm for selecting and determining a single molecular marker for the simple and reliable species delineation and phylogenetic inference of other bacterial taxa.

## Data Availability Statement

The datasets presented in this study can be found in online repositories. The names of the repository/repositories and accession number(s) can be found in the article/Supplementary Material.

## Author Contributions

YL and HZ: conceptualization and project administration. YL: data curation, software, visualization, and writing – original draft. YL and TP: formal Analysis and methodology. XZ, XD, M-RD, and HZ: funding acquisition. YL, TP, SY, JD, XZ, and XD: investigation. HZ: resources and supervision. YL, TP, and QY: validation. YL, QY, and HZ: writing – review and editing. All authors contributed to the article and approved the submitted version.

## Conflict of Interest

The authors declare that the research was conducted in the absence of any commercial or financial relationships that could be construed as a potential conflict of interest.

## Publisher’s Note

All claims expressed in this article are solely those of the authors and do not necessarily represent those of their affiliated organizations, or those of the publisher, the editors and the reviewers. Any product that may be evaluated in this article, or claim that may be made by its manufacturer, is not guaranteed or endorsed by the publisher.

## Footnotes

1
https://lpsn.dsmz.de/family/myxococcaceae

2
https://www.lynnon.com/

3
https://cran.r-project.org/web/packages/basicTrendline/index.html

4
https://lpsn.dsmz.de/species/myxococcus-macrosporus
