# Supplementary material for: Phylogenomic Analysis Substantiates the gyrB Gene as a Powerful Molecular Marker to Efficiently Differentiate the Most Closely Related Genera Myxococcus, Corallococcus, and Pyxidicoccus
Source: Front Microbiol. 2021 Oct 11;12:763359. doi: 10.3389/fmicb.2021.763359 (PMC8542856; doi:10.3389/fmicb.2021.763359)
Supplement: Supplementary file 1 [file Data_Sheet_1.PDF]

Phylogenomic analysis substantiates the *gyrB* gene as a powerful molecular marker to efficiently differentiate the most closely related genera *Myxococcus*, *Corallococcus*, and *Pyxidicoccus*

Yang Liu[1], Tao Pei[1], Shuoxing Yi[1], Juan Du[1], Xianjiao Zhang[1], Xiaoqin Deng[1], Qing Yao[2], Ming-Rong Deng[1], Honghui Zhu[1, *]

[1] Guangdong Provincial Key Laboratory of Microbial Culture Collection and Application, Key Laboratory of Agricultural Microbiomics and Precision Application, Ministry of Agriculture and Rural Affairs, State Key Laboratory of Applied Microbiology Southern China, Guangdong Open Laboratory of Applied Microbiology, Guangdong Microbial Culture Collection Center (GDMCC), Institute of Microbiology, Guangdong Academy of Sciences, Guangzhou 510070, P. R. China

[2] College of Horticulture, South China Agricultural University, Guangdong Province Key Laboratory of Microbial Signals and Disease Control, Guangzhou 510642, P. R. China

*Corresponding author: Honghui Zhu, E-mail: zhuhh@gdim.cn.

Supplementary materials

Supplementary Figures S1-S5

Figure S1 The heatmap of dDDH hybridization values between 90 genome sequences

Figure S1

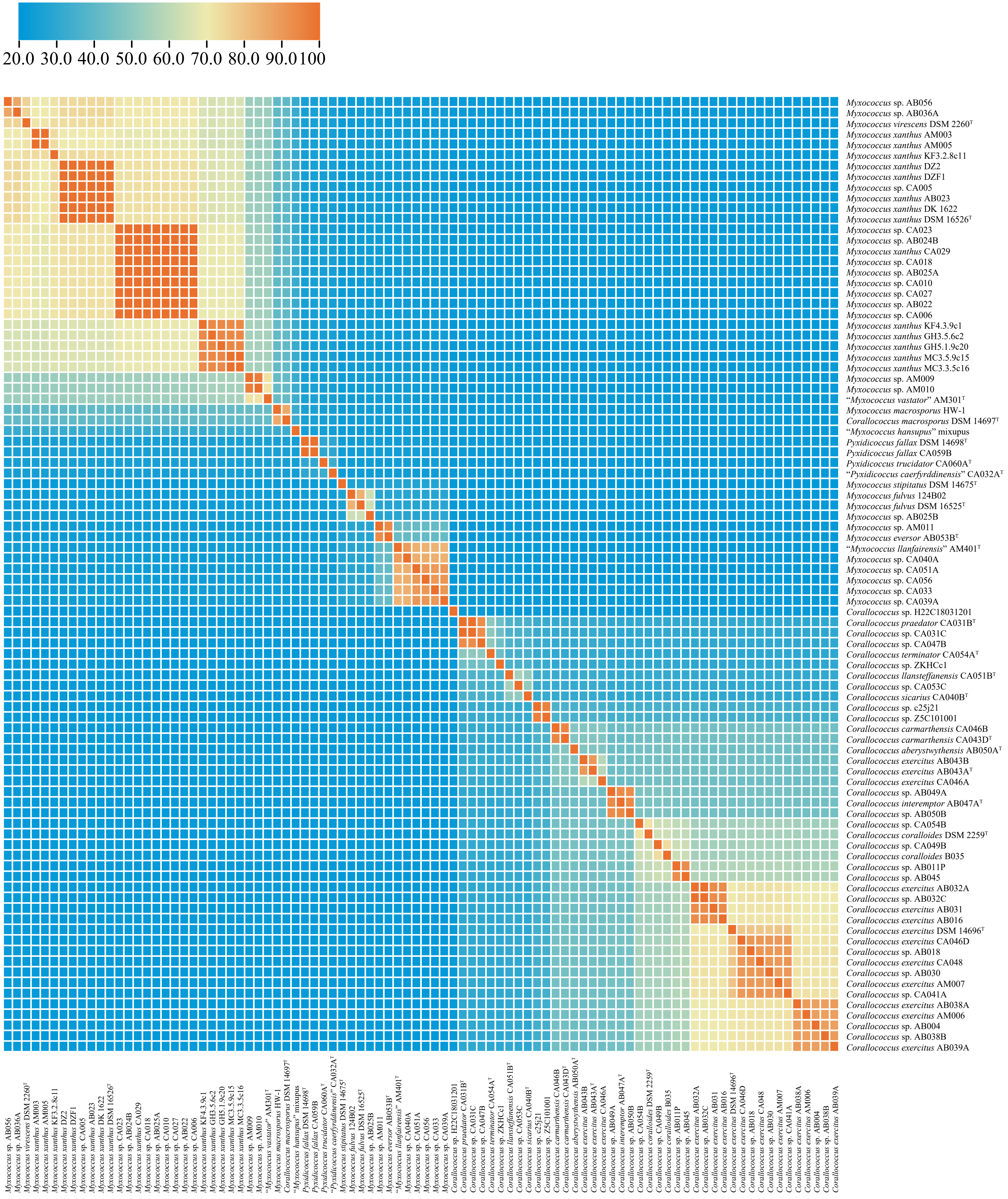

20.0 30.0 40.0 50.0 60.0 70.0 80.0 90.0 100
Myxococcus sp. AB056
Myxococcus sp. AB036A
Myxococcus virescens DSM 2260^{T}
Myxococcus xanthus AM003
Myxococcus xanthus AM005
Myxococcus xanthus KF3.2.8c11
Myxococcus xanthus DZ2
Myxococcus xanthus DZF1
Myxococcus sp. CA005
Myxococcus xanthus AB023
Myxococcus xanthus DK 1622
Myxococcus xanthus DSM 16526^{T}
Myxococcus sp. CA023
Myxococcus sp. AB024B
Myxococcus xanthus CA029
Myxococcus sp. CA018
Myxococcus sp. AB025A
Myxococcus sp. CA010
Myxococcus sp. CA027
Myxococcus sp. AB022
Myxococcus sp. CA006
Myxococcus xanthus KF4.3.9c1
Myxococcus xanthus GH3.5.6c2
Myxococcus xanthus GH5.1.9c20
Myxococcus xanthus MC3.5.9c15
Myxococcus xanthus MC3.3.5c16
Myxococcus sp. AM009
Myxococcus sp. AM010
"*Myxococcus vastator*" AM301^{T}
Myxococcus macrosporus HW-1
Corallococcus macrosporus DSM 14697^{T}
"*Myxococcus hansupus*" mixupus
Pyxidicoccus fallax DSM 14698^{T}
Pyxidicoccus fallax CA059B
Pyxidicoccus trucidator CA060A^{T}
"*Pyxidicoccus caerfyrddinensis*" CA032A^{T}
Myxococcus stipitatus DSM 14675^{T}
Myxococcus fulvus 124B02
Myxococcus fulvus DSM 16525^{T}
Myxococcus sp. AB025B
Myxococcus sp. AM011
Myxococcus eversor AB053B^{T}
"*Myxococcus llanfairensis*" AM401^{T}
Myxococcus sp. CA040A
Myxococcus sp. CA051A
Myxococcus sp. CA056
Myxococcus sp. CA033
Myxococcus sp. CA039A
Corallococcus sp. H22C18031201
Corallococcus praedator CA031B^{T}
Corallococcus sp. CA031C
Corallococcus sp. CA047B
Corallococcus terminator CA054A^{T}
Corallococcus sp. ZKHCc1
Corallococcus llansteffanensis CA051B^{T}
Corallococcus sp. CA053C
Corallococcus sicarius CA040B^{T}
Corallococcus sp. c25j21
Corallococcus sp. Z5C101001
Corallococcus carmarthensis CA046B
Corallococcus carmarthensis CA043D^{T}
Corallococcus aberystwythensis AB050A^{T}
Corallococcus exercitus AB043B
Corallococcus exercitus AB043A^{T}
Corallococcus exercitus CA046A
Corallococcus sp. AB049A
Corallococcus interemptor AB047A^{T}
Corallococcus sp. AB050B
Corallococcus sp. CA054B
Corallococcus coralloides DSM 2259^{T}
Corallococcus sp. CA049B
Corallococcus coralloides B035
Corallococcus sp. AB011P
Corallococcus sp. AB045
Corallococcus exercitus AB032A
Corallococcus sp. AB032C
Corallococcus exercitus AB031
Corallococcus exercitus AB016
Corallococcus exercitus DSM 14696^{T}
Corallococcus exercitus CA046D
Corallococcus sp. AB018
Corallococcus exercitus CA048
Corallococcus sp. AB030
Corallococcus exercitus AM007
Corallococcus sp. CA041A
Corallococcus exercitus AB038A
Corallococcus exercitus AM006
Corallococcus sp. AB004
Corallococcus sp. AB038B
Corallococcus exercitus AB039A

Figure S2 The violin plots of intraspecies and interspecies identity distributions of the complete and 966-bp *gyrB* gene sequences and the complete 16S rRNA gene sequences. The lengths of the complete *gyrB* and 16S rRNA gene sequences used in this analysis were 2448-2466 and 1536-1538 bp, respectively.

Figure S2

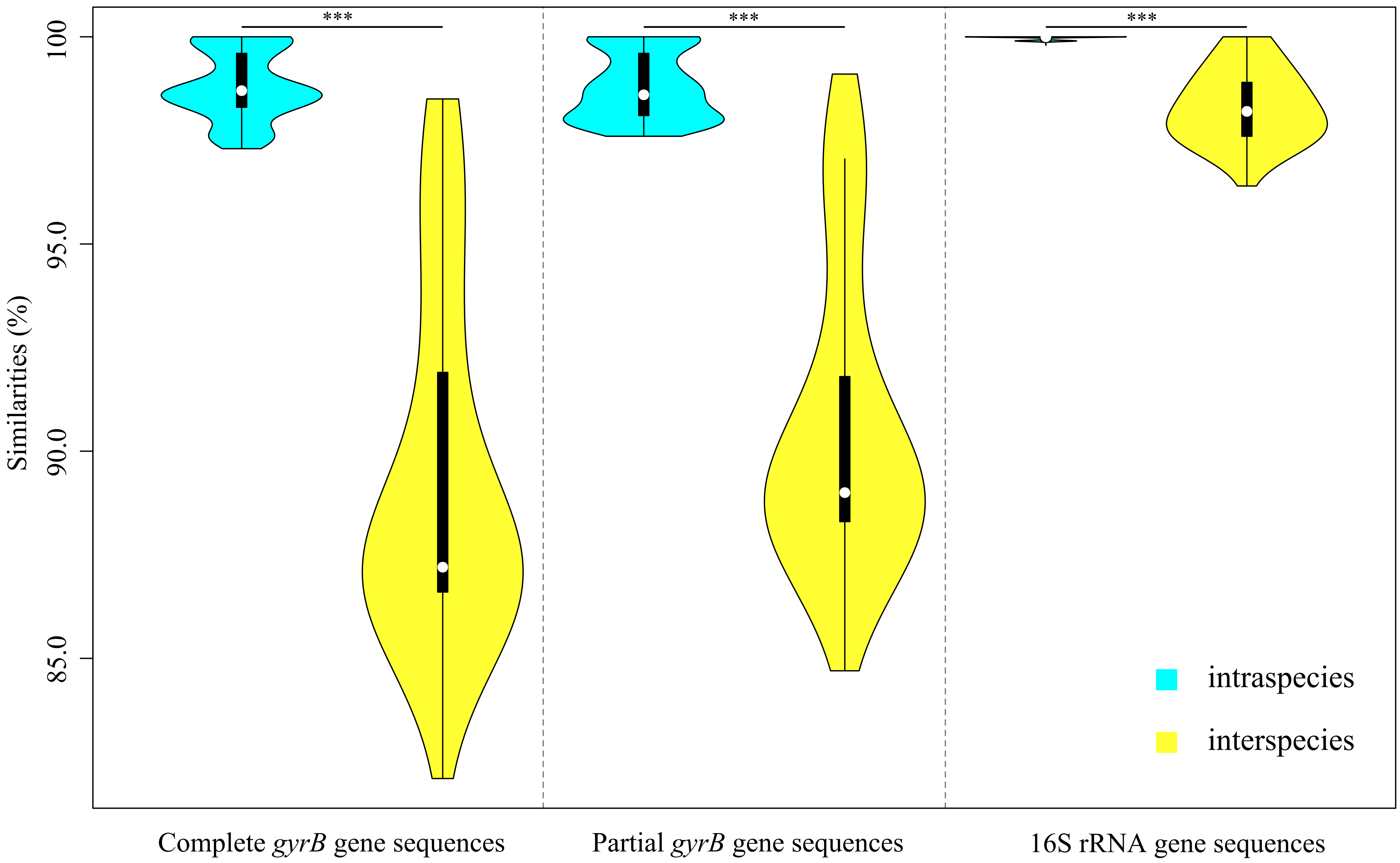

Similarities (%)
100
95.0
90.0
85.0
***
***
***
intraspecies
interspecies
Complete *gyrB* gene sequences
Partial *gyrB* gene sequences
16S rRNA gene sequences

Figure S3 Gel image of PCR products of three representative isolates

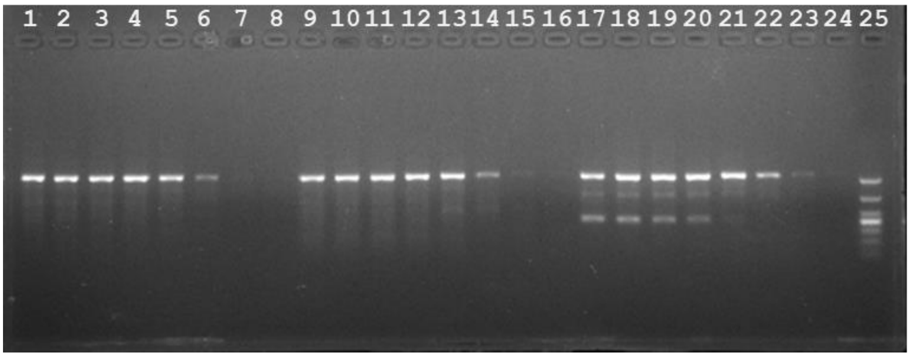

(1) Lanes 1, 9, and 17, the annealing temperature was setted on 56 ºC; lanes 2, 10, and 18, 56.7 ºC; lanes 3, 11, and 19, 57.9 ºC; lanes 4, 12, and 20, 59.8 ºC; lanes 5, 13, and 21, 62.1 ºC; lanes 6, 14, and 22, 64.0 ºC; lanes 7, 17, and 23, 65.3 ºC; lanes 8, 16, and 26, 66.0 ºC.

(2) Lanes 1-8, isolate AS-1-12; lanes 9-16, isolate XM-1-8; lanes 17-24, isolate RHSTA-1-4; lane 25, the 1 kb marker.

Figure S4 The maximum-likelihood (ML) phylogenetic tree based on 966-bp *gyrB* gene sequences. The ML tree of the 966-bp *gyrB* gene sequences was reconstructed using the IQ-TREE with the TIM + F + I + G4 model. The species names are effectively but not yet validly published and thus are in quotation marks. Strain *Aggregicoccus* sp. 17bor-14 was used as an outgroup. Bootstrap values great than 80% were shown at branch points. Bar: 0.05 represents the number of substitutions per site.

Figure S4

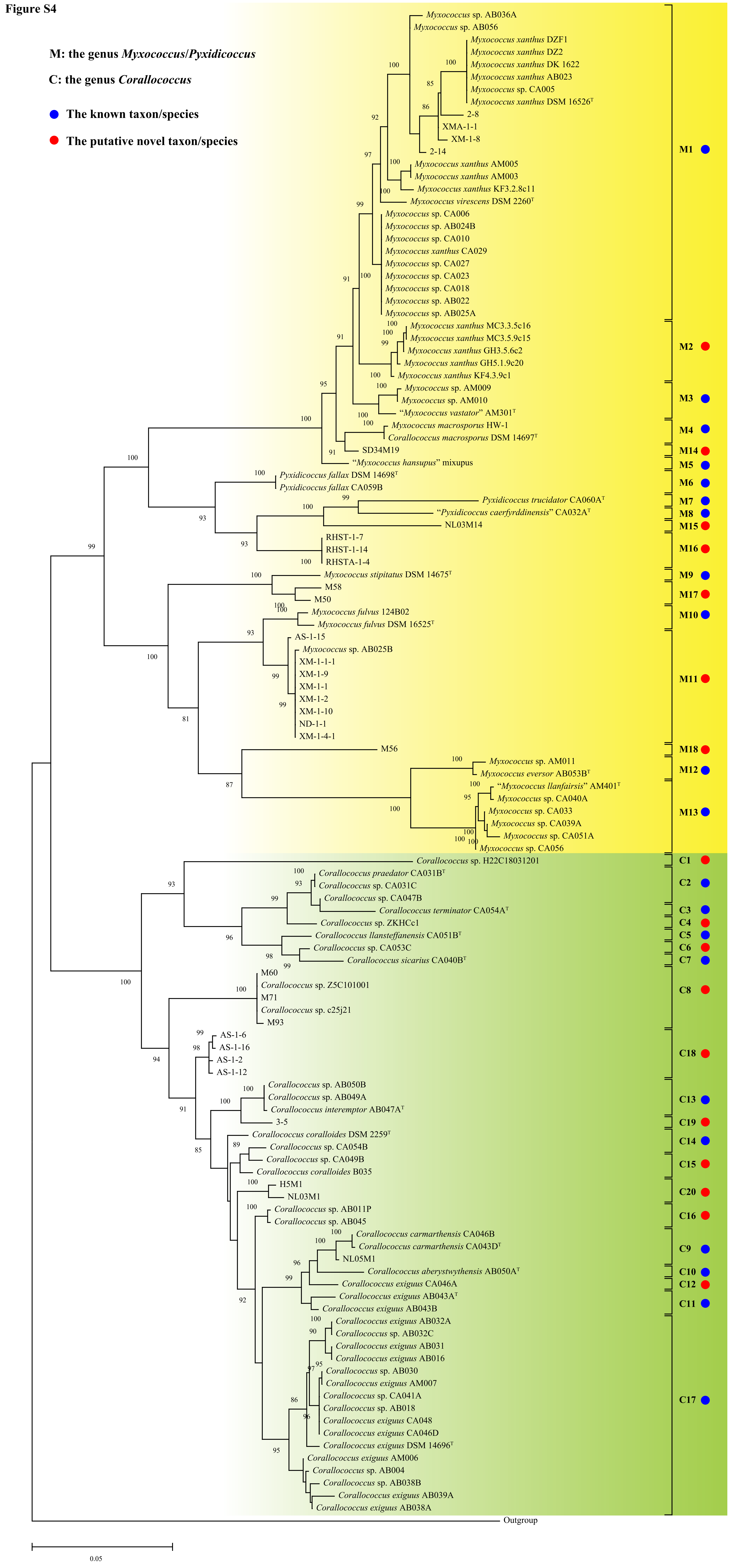
M: the genus Myxococcus/Pyxidicoccus
C: the genus Corallococcus
The known taxon/species
The putative novel taxon/species
Myxococcus sp. AB036A
Myxococcus sp. AB056
Myxococcus xanthus DZF1
Myxococcus xanthus DZ2
Myxococcus xanthus DK 1622
Myxococcus xanthus AB023
Myxococcus sp. CA005
Myxococcus xanthus DSM 16526T
2-8
XMA-1-1
XM-1-8
2-14
Myxococcus xanthus AM005
Myxococcus xanthus AM003
Myxococcus xanthus KF3.2.8c11
Myxococcus virescens DSM 2260T
Myxococcus sp. CA006
Myxococcus sp. AB024B
Myxococcus sp. CA010
Myxococcus xanthus CA029
Myxococcus sp. CA027
Myxococcus sp. CA023
Myxococcus sp. CA018
Myxococcus sp. AB022
Myxococcus sp. AB025A
Myxococcus xanthus MC3.3.5c16
Myxococcus xanthus MC3.5.9c15
Myxococcus xanthus GH3.5.6c2
Myxococcus xanthus GH5.1.9c20
Myxococcus xanthus KF4.3.9c1
Myxococcus sp. AM009
Myxococcus sp. AM010
"Myxococcus vastator" AM301T
Myxococcus macrosporus HW-1
Corallococcus macrosporus DSM 14697T
SD34M19
"Myxococcus hansupus" mixupus
Pyxidicoccus fallax DSM 14698T
Pyxidicoccus fallax CA059B
Pyxidicoccus trucidator CA060AT
"Pyxidicoccus caerfyrddinensis" CA032AT
NL03M14
RHST-1-7
RHST-1-14
RHSTA-1-4
Myxococcus stipitatus DSM 14675T
M58
M50
Myxococcus fulvus 124B02
Myxococcus fulvus DSM 16525T
AS-1-15
Myxococcus sp. AB025B
XM-1-1-1
XM-1-9
XM-1-1
XM-1-2
XM-1-10
ND-1-1
XM-1-4-1
M56
Myxococcus sp. AM011
Myxococcus eversor AB053BT
"Myxococcus llanfairsis" AM401T
Myxococcus sp. CA040A
Myxococcus sp. CA033
Myxococcus sp. CA039A
Myxococcus sp. CA051A
Myxococcus sp. CA056
Corallococcus sp. H22C18031201
Corallococcus praedator CA031BT
Corallococcus sp. CA031C
Corallococcus sp. CA047B
Corallococcus terminator CA054AT
Corallococcus sp. ZKHCc1
Corallococcus llansteffanensis CA051BT
Corallococcus sp. CA053C
Corallococcus sicarius CA040BT
M60
Corallococcus sp. Z5C101001
M71
Corallococcus sp. c25j21
M93
AS-1-6
AS-1-16
AS-1-2
AS-1-12
Corallococcus sp. AB050B
Corallococcus sp. AB049A
Corallococcus interemptor AB047AT
3-5
Corallococcus coralloides DSM 2259T
Corallococcus sp. CA054B
Corallococcus sp. CA049B
Corallococcus coralloides B035
H5M1
NL03M1
Corallococcus sp. AB011P
Corallococcus sp. AB045
Corallococcus carmarthensis CA046B
Corallococcus carmarthensis CA043DT
NL05M1
Corallococcus aberystwythensis AB050AT
Corallococcus exiguus CA046A
Corallococcus exiguus AB043AT
Corallococcus exiguus AB043B
Corallococcus exiguus AB032A
Corallococcus sp. AB032C
Corallococcus exiguus AB031
Corallococcus exiguus AB016
Corallococcus sp. AB030
Corallococcus exiguus AM007
Corallococcus sp. CA041A
Corallococcus sp. AB018
Corallococcus exiguus CA048
Corallococcus exiguus CA046D
Corallococcus exiguus DSM 14696T
Corallococcus exiguus AM006
Corallococcus sp. AB004
Corallococcus sp. AB038B
Corallococcus exiguus AB039A
Corallococcus exiguus AB038A
Outgroup
M1
M2
M3
M4
M14
M5
M6
M7
M8
M15
M16
M9
M17
M10
M11
M18
M12
M13
C1
C2
C3
C4
C5
C6
C7
C8
C18
C13
C19
C14
C15
C20
C16
C9
C10
C12
C11
C17
0.05

Figure S5 Correlation analysis between dDDH and the complete *gyrB* gene sequences identities. Correlation analysis was simulated using the "exp3P" model of the "basicTrendline" library in R.

Figure S5

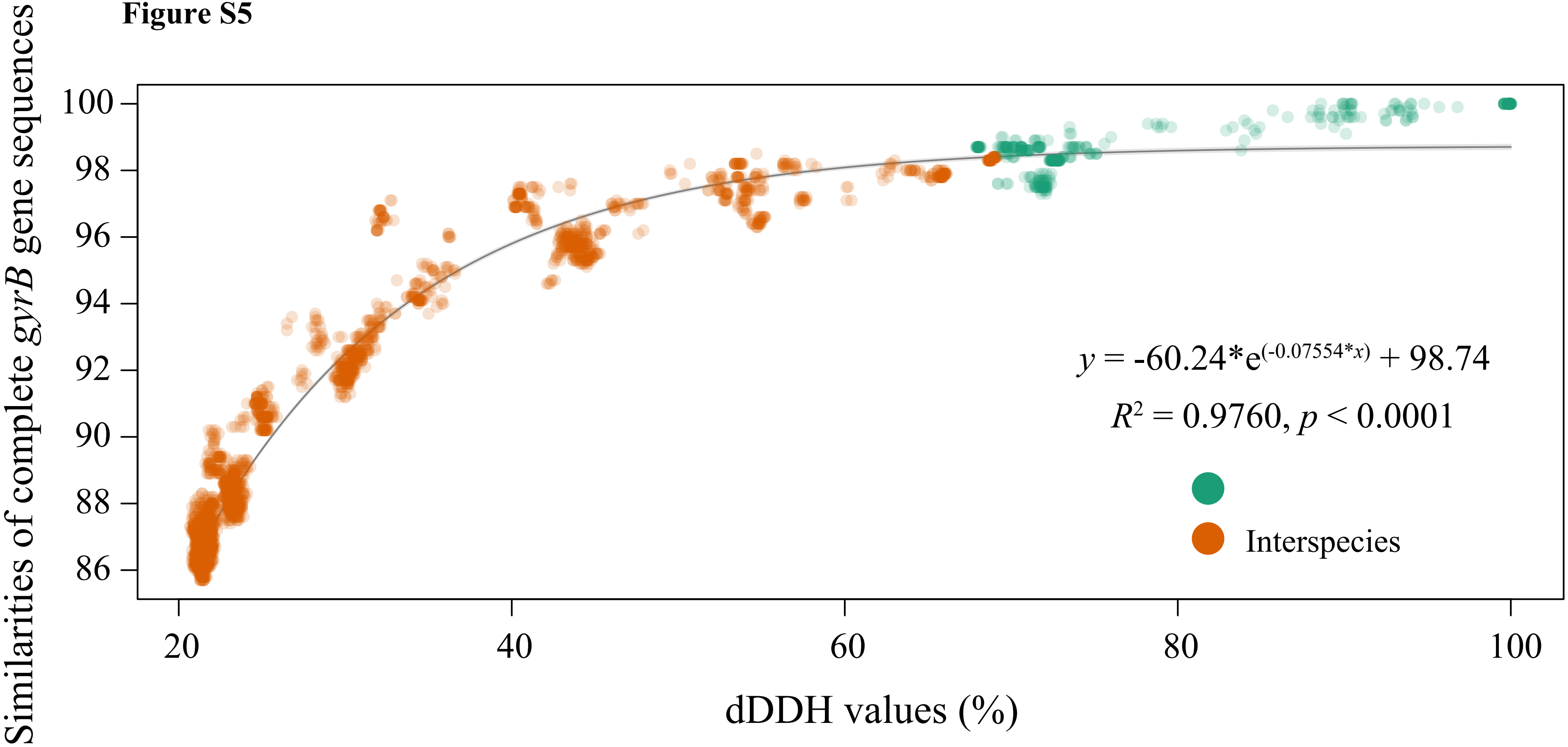
Similarities of complete gyrB gene sequences
100
98
96
94
92
90
88
86
20
40
60
80
100
dDDH values (%)
y = -60.24*e^(-0.07554*x) + 98.74
R^2 = 0.9760, p < 0.0001
Interspecies

Supplementary Tables S1-S9

Table S1 The detailed information on 31 isolates

Table S2 The 1887 core genes for the representative model myxobacterium *Myxococcus xanthus* DK1622

Table S3 The pairwise dDDH values between 90 genome sequences

Table S4 The pairwise identities of complete *gyrB* gene sequences from 90 genome sequences used in this study

Table S5 The pairwise identities of complete 16S rRNA gene sequences from 90 genome sequences used in this study

Table S6 The pairwise identities of 966-bp *gyrB* gene sequences from 90 genome sequences used in this study

Table S7 The pairwise identities of 966-bp *gyrB* gene sequences from 90 genome sequences and 31 isolates used in this study

Table S8 The information on genome sequences of the five representative isolates

Table S9 The pairwise dDDH values between each of the five representative isolates and reference genome sequences
